# When the Blisters Reach the Heart: An Uncommon Case of Cardiac Involvement in Bullous Pemphigoid

**DOI:** 10.7759/cureus.68124

**Published:** 2024-08-29

**Authors:** Jagadeswar Kakumani, Prem Balaji Reddy Lankapothu, Amukthamalyada Koduri, Magesh Kumar S

**Affiliations:** 1 Internal Medicine, Saveetha Medical College and Hospitals, Saveetha Institute of Medical and Technical Sciences, Saveetha University, Chennai, IND

**Keywords:** bradyarrhythmias, blisters, autoimmune skin disorders, bullous dermatoses, complete heart block

## Abstract

Bullous pemphigoid is an autoimmune blistering disorder predominantly affecting the elderly, with rare systemic complications, including cardiac involvement. This case study presents a 46-year-old female with a history of arterial hypertension who developed complete heart block (CHB) associated with bullous pemphigoid. The patient initially presented with bilateral lower limb swelling and discoloration, later diagnosed as bullous pemphigoid following the appearance of characteristic skin lesions. Concurrently, she was found to have asymptomatic bradycardia, with an electrocardiogram confirming CHB. After ruling out other causes of heart block, a dual-chamber pacemaker was implanted, and the patient was treated with immunosuppressive therapy to control the autoimmune blistering disorder. This case highlights the rare but significant association between bullous pemphigoid and CHB, emphasizing the importance of multidisciplinary care and timely intervention in managing such complex cases.

## Introduction

Bullous pemphigoid is a rare autoimmune blistering disorder that primarily affects elderly individuals and is characterized by large, fluid-filled blisters on areas of the skin that often flex, such as the lower abdomen, upper thighs, and armpits [1]. The pathogenesis involves autoantibodies against hemidesmosomal proteins in the epidermal basement membrane, leading to subepidermal blister formation. While bullous pemphigoid is primarily a skin disorder, it is occasionally associated with systemic manifestations, though cardiovascular involvement is quite rare [2]. Common systemic manifestations of bullous pemphigoid are stroke, myocardial infarction, and other thrombotic phenomena. However, conduction block is a very rare association. Complete heart block (CHB) is a life-threatening arrhythmia characterized by a complete dissociation between atrial and ventricular activity, leading to bradycardia and potentially severe hemodynamic compromise [3]. The etiology of CHB is diverse, ranging from ischemic heart disease to infiltrative processes and iatrogenic causes. However, the association between bullous pemphigoid and CHB is extraordinarily uncommon, and the causal relationship remains uncertain, making this case particularly noteworthy [4]. This case study explores the clinical presentation, diagnostic process, and management of a 46-year-old female patient with CHB in the context of bullous pemphigoid, highlighting the diagnostic challenges and therapeutic decisions involved.

## Case presentation

A 46-year-old female with a known history of systemic hypertension on treatment presented with bilateral lower limb swelling and discoloration of 15 days’ duration. She was initially treated for cellulitis at a local hospital, where she was incidentally found to have bradycardia, with a heart rate of 40-50 beats per minute, despite the absence of cardiac-related symptoms. Due to these abnormal findings, she was referred to our facility for further evaluation. Upon admission, an electrocardiogram (ECG) confirmed the presence of CHB. Notably, the patient also had visible skin lesions, leading to consultations with both dermatology and cardiology specialists.

**Figure 1:**
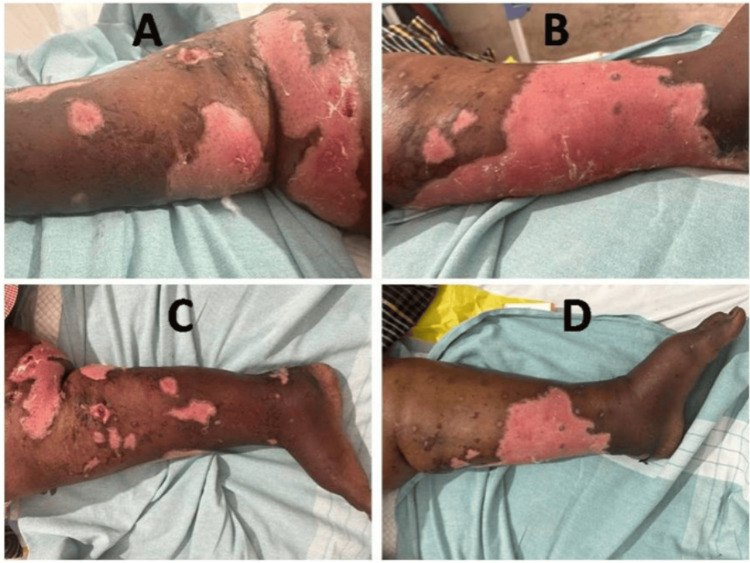
(A) Extensive erosions. (B) A broad area of denuded skin with clear demarcation between the affected and unaffected skin. The eroded areas are extensive, and there are some intact bullae visible on the surrounding skin. (C) The entire lower limb with multiple lesions at various stages. Some areas are fully denuded, others show hyperpigmented scars, and there are active bullae and erosions. (D) The lateral aspect of the lower leg and foot, showing a large, continuous area of denuded skin. There are also some smaller, intact bullae visible.

Figure 1A illustrates the active and severe stage of bullous pemphigoid, where large bullae have ruptured, leaving behind extensive erosions vulnerable to infection. Figure 1B highlights the aggressive nature of the disease in this patient. Figure 1C demonstrates the widespread and multifocal nature of the lesions, indicating the chronicity and severity of the bullous pemphigoid in this patient. Figure 1D depicts the extent of skin damage and the risk of secondary complications, such as infection, due to the large areas of exposed skin. Large, tense bullae on erythematous skin, usually seen in the elderly but can occur in young, with minimal mucosal involvement, ruled out dermatitis herpetiformis, linear IgA dermatosis, and epidermolysis bullosa acquisita which have distinct clinical features such as grouped vesicles, linear patterns, and trauma-induced blister. Immunofluorescence and enzyme-linked immunosorbent assay revealed that the skin lesions were consistent with a diagnosis of bullous pemphigoid. ECG showed CHB with a narrow QRS escape rhythm with a heart rate of 48 beats per minute with no significant ST and T changes. A two-dimensional echocardiogram was normal. An extensive workup was performed to rule out common etiologies of CHB, including ischemic heart disease, myocarditis, and electrolyte imbalances, all of which were negative. The temporal proximity between the onset of bullous pemphigoid and the development of CHB suggested a potential causal relationship between the two conditions. Due to the risk of progression to symptomatic bradycardia and potential asystole, the patient was considered a suitable candidate for pacemaker implantation. A dual-chamber pacemaker was successfully implanted without complications, ensuring both atrioventricular synchrony and adequate heart rate support. The patient was also initiated on Doxycycline 100 mg, Momate-F cream, saline compresses BD, and oral Prednisone 1 mg/kg to manage the bullous pemphigoid. Her postoperative course was uneventful, with a resolution of bradycardia and marked improvement in skin lesions.

## Discussion

The relationship between autoimmune diseases and cardiac conduction abnormalities is well-documented, particularly in conditions such as systemic lupus erythematosus and rheumatoid arthritis [5,6]. However, the development of CHB in the context of bullous pemphigoid is rare and not well understood [7]. It is hypothesized that the inflammatory milieu in bullous pemphigoid, characterized by elevated circulating autoantibodies and immune complexes, may contribute to myocardial inflammation or direct involvement of the conduction system, ultimately leading to CHB [8,9]. The study on endemic pemphigus foliaceous in El Bagre, Colombia, reported several cardiac arrhythmias, including sinus bradycardia, left bundle branch block, left posterior fascicular block, and left anterior fascicular block [10]. These findings suggest a potential link between autoantibody deposition in the cardiac conduction system and arrhythmias, which may be relevant to understanding the rare occurrence of CHB in our patient with bullous pemphigoid [10]. The management of CHB necessitates prompt recognition and intervention, often requiring pacemaker implantation to mitigate the risks associated with bradycardia and potential sudden cardiac death [11,12]. In this case, the decision to implant a pacemaker was based on the confirmed presence of CHB and the exclusion of reversible causes. Concomitant treatment of bullous pemphigoid with immunosuppressive agents was also crucial to addressing the underlying autoimmune pathology.

## Conclusions

This case underscores the rare but critical hypothesis of a link between bullous pemphigoid and CHB, highlighting the need for awareness and vigilance in diagnosing and managing such complex presentations. While bullous pemphigoid is primarily recognized for its dermatological manifestations, the potential for systemic complications, including cardiac involvement, should not be overlooked. The identification of CHB in this patient prompted a comprehensive evaluation to exclude other common causes and allowed for timely intervention with a dual-chamber pacemaker. This early recognition and treatment were vital in addressing the bradycardia and preventing potential hemodynamic instability. Furthermore, the initiation of immunosuppressive therapy for bullous pemphigoid was essential in controlling the underlying autoimmune process and preventing further progression of both skin and systemic manifestations.

Future research should continue to explore the mechanisms linking autoimmune disorders with cardiac conduction abnormalities and establish guidelines for the management of such rare yet significant associations. This case contributes to the growing body of evidence underscoring the need for heightened clinical awareness and comprehensive management strategies in the treatment of patients with autoimmune conditions and associated systemic complications.
